# β-Casein Polymorphism in Serbian Holstein-Friesian and Busha Cattle and Its Association with Milk Production Traits

**DOI:** 10.3390/ani16132052

**Published:** 2026-07-03

**Authors:** Marko Ristanic, Uros Glavinic, Jevrosima Stevanovic, Jovana Stamenovic, Nemanja M. Jovanovic, Milan Maletic, Zoran Stanimirovic

**Affiliations:** 1 Department of Biology, Faculty of Veterinary Medicine, University of Belgrade, Bul. Oslobodjenja 18, 11000 Belgrade, Serbia; uglavinic@vet.bg.ac.rs (U.G.); rocky@vet.bg.ac.rs (J.S.); jovana.stamenovic@vet.bg.ac.rs (J.S.); zoran@vet.bg.ac.rs (Z.S.); 2 Department of Parasitology and Parasitic Diseases, Faculty of Veterinary Medicine, University of Belgrade, Bul. Oslobodjenja 18, 11000 Belgrade, Serbia; nmjovanovic@vet.bg.ac.rs; 3 Department of Reproduction, Fertility and Artificial Insemination, Faculty of Veterinary Medicine, University of Belgrade, Bul. Oslobodjenja 18, 11000 Belgrade, Serbia; maletic@vet.bg.ac.rs

**Keywords:** β-casein, A2 milk, Holstein, Busha, genotyping, milk production traits

## Abstract

Interest in β-casein polymorphism and A2 milk production has increased considerably in recent years because of their potential effects on milk quality and dairy cattle breeding. The aim of this study was to determine the distribution of β-casein A1/A2 genotypes in Holstein-Friesian (HF) cattle and indigenous Busha cattle from Serbia and to evaluate the association between β-casein genotypes and milk production traits in HF cows. The study included 334 HF cows, 58 HF bulls, and 64 Busha cows. Genotyping was performed using the ACRS-PCR method, followed by restriction fragment analysis. Milk production data for HF cows included milk yield, milk protein content, and milk fat content during the first and second lactation. Statistical analysis included determination of genotype and allele frequencies, testing of Hardy–Weinberg equilibrium, and evaluation of associations between genotypes and milk production traits. In HF cows, genotype frequencies were 14.07% for A1A1, 60.18% for A1A2, and 25.75% for A2A2, while allele frequencies were 0.442 for A1 and 0.558 for A2. Similar genotype and allele distributions were observed in HF bulls, where the A2 allele frequency (0.560) was slightly higher than the A1 allele frequency (0.440). In Busha cows, a pronounced predominance of the A2 allele (0.719) and A2A2 genotype (56.25%) was detected. HF cows carrying the A2A2 genotype were associated with significantly higher milk yield, as well as higher milk protein and milk fat content, compared to cows with other genotypes (*p* < 0.05; *p* < 0.01). Milk production traits were generally higher during the second lactation, regardless of genotype. HF cows carrying the A2A2 genotype generally showed higher milk yield and more favorable milk composition traits. In addition, the high frequency of the A2 allele in Busha cattle indicates that this indigenous breed may represent a valuable genetic resource for the conservation and utilization of the A2 allele in future breeding programs.

## Simple Summary

β-casein is one of the main milk proteins in cattle and occurs in several genetic variants, of which A1 and A2 are the most important and most studied. In recent years, increasing attention has been directed toward A2 milk because of growing scientific and commercial interest related to differences in β-casein composition and digestion. The aim of this study was to determine the distribution of A1 and A2 β-casein variants in Holstein-Friesian (HF) cattle and indigenous Busha cattle from Serbia and to investigate the relationship between β-casein genotypes and milk production traits in HF cows. The results showed a higher frequency of the A2 allele in both breeds, especially in Busha cattle, where the A2A2 genotype was dominant. In HF cows, animals carrying the A2A2 genotype achieved higher milk yield, as well as higher milk protein and milk fat content, compared to cows with other genotypes. These findings highlight the importance of β-casein polymorphism in dairy cattle breeding and emphasize the potential value of the indigenous Busha breed as a genetic resource for future breeding programs aimed at increasing the frequency of the A2-associated β-casein variant.

## 1. Introduction

Cattle breeding in Serbia has a long tradition, while animal husbandry has historically represented one of the most important branches of agriculture in the region. Indigenous cattle breeds such as Busha were traditionally bred in Serbian households. Busha belongs to the short-horned cattle group (*Bos brachyceros europaeus*) and is also known as domestic short-horned cattle. Until the middle of the twentieth century, Busha was the dominant cattle breed in the Balkans, after which it was gradually replaced by more productive breeds such as Simmental and Holstein-Friesian (HF), which are the most represented breeds today.

According to the Statistical Office of the Republic of Serbia, the total number of cattle in Serbia on 1 December 2024, was 698,605 animals, confirming the continuing long-term decline in livestock production [1]. Although official statistics do not provide breed-specific data, reports from the Institute for Animal Husbandry, Belgrade–Zemun [2], indicate that selection programs in 2024 included 11,614 registered HF cows, representing the basis of organized dairy production in Serbia. At the same time, conservation programs anticipate the inclusion of approximately 1400 Busha cows in breeding programs during 2025, confirming the importance of this breed as a protected genetic resource of national significance [3].

Indigenous cattle breeds are well adapted to local environmental conditions and are, therefore, important for extensive production systems. Improving knowledge about genetic diversity within and among indigenous breeds is important for their sustainable use and conservation [4]. Although indigenous breeds are generally characterized by dual-purpose production, Busha cattle in Serbia are predominantly bred for meat production rather than dairy production, mainly due to poor infrastructure and high feeding costs. Nevertheless, Busha cows produce approximately 800 L of milk during lactation [5].

The average milk yield per cow in Serbia remains considerably lower than that reported in most European Union countries, reflecting differences in breed structure, farm size, management practices, and production intensity. Average annual milk production per cow in Serbia is estimated at 3500–4500 L, depending on production intensity, herd genetics, and feeding conditions, which is still considerably lower than in most developed European countries, where production frequently exceeds 7000 L per cow annually [6]. All of these findings indicate that additional genetic improvement and more targeted selection strategies are still needed.

In recent years, more attention has been directed toward milk quality traits, especially β-casein polymorphism and A2 milk production. Cow’s milk contains four main casein groups, αs1-, αs2-, β-, and κ-casein, which account for approximately 38%, 10%, 36%, and 13% of total milk proteins, respectively [7]. Among them, β-casein is one of the most polymorphic milk proteins, with at least 15 identified variants, including A1, A2, A3, B, C, D, E, F, G, H1, H2, I, J, K, and L [8]. Although several additional β-casein variants have been described, the A1 and A2 variants are the most common in Holstein-Friesian cattle and, therefore, represent the primary focus of most studies investigating β-casein polymorphism [8,9].

**Figure 1:**
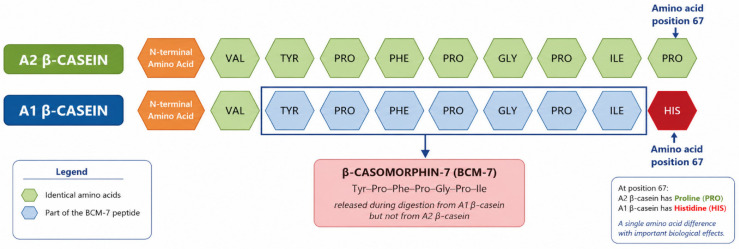
Difference between A1 and A2 β-casein variants and creation of beta-casomorphine-7 (BCM-7).

The difference between A1 and A2 β-casein results from a single amino acid substitution at position 67 of the polypeptide chain, where histidine is present in the A1 variant, and proline in the A2 variant (Figure 1). This substitution is caused by a nucleotide change from cytosine (C) to adenine (A) in the β-casein gene, causing a codon change from CCT to CAT, and, consequently, replacing proline with histidine at position 67 (Figure 1).

During digestion, the presence of histidine at position 67 in A1 β-casein may facilitate the release of β-casomorphin-7 (BCM-7), a bioactive opioid peptide, whereas the release of BCM-7 from A2 β-casein is considerably reduced. Although several studies [10,11,12,13,14,15,16] have investigated possible associations between BCM-7 and gastrointestinal, inflammatory, or other physiological responses, the biological significance of these findings and their implications for human health remain a subject of ongoing scientific debate.

Previous studies suggest that the A2 allele represents the ancestral form of β-casein, while the A1 allele likely originated later through mutation during cattle domestication [17,18]. The frequency of A1 and A2 alleles varies between breeds and geographic regions. Research conducted in different countries indicates that the A1 allele is dominant in Northern European cattle populations, whereas the frequency of the A2 allele tends to increase in Central and Southern European breeds [19]. In Serbia, limited studies have investigated β-casein polymorphism in dairy cattle populations, particularly in indigenous breeds. Ristanic et al. [20] reported β-casein genotype frequencies in HF cattle and suggested possible associations between the A2 allele and improved milk production traits. In Supplementary Table S1 [4,19,20,21,22,23,24,25,26,27,28,29,30,31,32,33,34,35,36,37,38,39,40,41,42,43,44,45,46,47], data on β-casein allele frequencies and genotypes in HF and autochthonous cattle—conducted in several countries worldwide—are presented.

The CSN2 gene, which encodes β-casein, is located within the casein gene cluster on bovine chromosome 6 (BTA6). Due to its biological role in milk protein synthesis and its association with variation in milk composition traits, CSN2 is considered one of the most important candidate genes affecting milk quality in dairy cattle [12,48]. Recent genomic studies also showed that polymorphisms within the β-casein gene contribute significantly to variation in milk composition traits and dairy production performance [49]. Growing interest in A2 milk has increased the importance of β-casein genotyping in dairy cattle breeding. To date, information regarding β-casein polymorphism in Serbian cattle populations remains limited, particularly for indigenous breeds and breeding bulls.

The aim of this study was to characterize β-casein polymorphism in commercial and indigenous cattle populations in Serbia and to evaluate its association with milk production traits in Holstein-Friesian cows. The study included Holstein-Friesian cows, Holstein-Friesian bulls, and indigenous Busha cattle, thereby enabling a broader population-genetic assessment of CSN2 variability than previously available. In addition to investigating genotype–phenotype associations in dairy cattle, this study provides the first data on β-casein polymorphism in Serbian Busha cattle.

## 2. Materials and Methods

### 2.1. Ethics Statement

All procedures involving animals were conducted in accordance with the national legislation governing animal welfare and scientific research. The study was approved by the Ethics Council of the Veterinary Directorate, Ministry of Agriculture, Forestry and Water Management of the Republic of Serbia, Decision No: 323-07-06299/2018-05, issued on 23 July 2018.

### 2.2. Animals Included in the Study and Sample Collection

Bulls: The material used for DNA extraction and subsequent molecular-genetic analyses was frozen semen from 58 bulls of the HF breed from the artificial insemination center of the Al Dahra Corporation (formerly PKB Corporation, Belgrade, Serbia). Semen samples were transported to the laboratory in a liquid-nitrogen container at a temperature of −196 °C and were stored under the same conditions until DNA extraction.

Cows: The material used for DNA extraction and subsequent molecular-genetic analyses was blood collected from 334 cows of the HF breed from farms of the Al Dahra Corporation (formerly PKB Corporation)—all HF cows were kept under the same conditions, in a tied housing system, and fed the same diet. Samples were also collected from 64 indigenous cattle of the Busha breed, from breeding sites on Stara Planina in Eastern Serbia—all Busha cattle were reared under the same extensive production conditions in a free-range grazing system and were exposed to similar environmental and feeding conditions. Inclusion in the study for HF cows required the availability of complete production records for both the first and second lactations. Therefore, only cows that had completed their second lactation were included. Meanwhile, their age distribution was relatively uniform, with most cows being approximately 4–5 years old at the time of sampling. Blood was collected aseptically by puncture of the tail vein into 10 mL vacutainers containing the anticoagulant EDTA and stored at 4 °C until the DNA extraction process. All blood was collected as part of routine activities within the Animal Health Protection Program, during the period between 2016 and 2021.

A subset of the Holstein-Friesian cows included in the present study (n = 106) was previously analyzed in a preliminary investigation of β-casein polymorphism in Serbian Holstein-Friesian cattle [20]. In the current study, the dataset was substantially expanded to include a total of 334 Holstein-Friesian cows, 58 Holstein-Friesian bulls, and 64 indigenous Busha cattle. The enlarged sample size enabled a more robust assessment of CSN2 allele and genotype frequencies and allowed for comparisons between commercial and indigenous cattle populations.

### 2.3. Collection of Data on Milk Yield, Milk Protein Content, and Milk Fat Content in HF Cows

Data on protein content, milk fat content, and milk yield of the observed cows were obtained directly from the database of the respective farms of the Al Dahra Corporation (formerly PKB Corporation). Data were collected from 334 HF cows for the complete first two lactations, including: total milk yield; total milk protein content; total milk fat content; milk yield corrected to 305 days; milk protein content corrected to 305 days; and milk fat content corrected to 305 days. The milk production data used in this study were collected between 2016 and 2021. After genotyping and analyzing allele and genotype frequencies for 334 HF cows, statistical processing was performed for the collected data on milk yield, milk protein content, and milk fat content of the first two lactations, and these results were mutually compared. Milk yield and the content of milk protein and milk fat in cows with different β-casein genotypes were compared in two ways: A—within the first and second lactations, observed separately; B—within the same genotype group, between the first and second lactations. Additionally, the mean values of the aforementioned parameters were compared, taking into account production across both lactations combined.

### 2.4. DNA Extraction

DNA extraction was performed using the commercial BLOOD Version II kit (Lucigen Corporation, Middleton, WI, USA) following the protocol of the Laboratory for Animal Production and Health, International Atomic Energy Agency—IAEA (Seibersdorf, Vienna, Austria), which was further adapted for the purposes of this study. Briefly, DNA was extracted from 1 mL of whole blood and involved the removal of erythrocytes, lysis of white blood cells, and precipitation and pelleting of genomic DNA. The extracted DNA samples were quantified using a UV–Vis spectrophotometer (BioSpec-nano, Shimadzu Scientific Instruments, Kyoto, Japan) to assess DNA quality.

### 2.5. ACRS-PCR

A 321 bp fragment was amplified using the ACRS-PCR method (Amplification Created Restriction Site-ACRS-PCR). The PCR-ACRS assay targets the nucleotide polymorphism responsible for the proline-to-histidine substitution in β-casein, which is commonly used for discrimination between the A1- and A2-type β-casein variants in dairy cattle. The reverse primer sequence was identical for the detection of both alleles, while the forward primer was designed so that the penultimate base at the 3′ end would be substituted with cytosine in the presence of the A1 allele, thereby creating a restriction site. Due to the difference in this single base pair between A1 and A2 β-casein, the endonuclease enzyme recognizes the specified restriction site and cuts the DNA product of the A1A1 genotype into two fragments, which appear as two bands on the electrophoretic gel (284 and 37 bp) (Supplementary Figure S1). Since this restriction site is not created in the A2 allele, only a single uncut band of 321 bp was observed for the A2A2 genotype. For the A1A2 genotype, three bands were observed on the gel at 321, 284, and 37 bp (Supplementary Figure S1).

The analysis was performed using a Bio-Rad T100 Thermal Cycler (Bio-Rad, Hercules, CA, USA). PCR was performed using the following primers: CASB forward: 5′ GCAGAATTCTAGTCTATCCCTTCCCTGGACCCATGC 3′ and CASB reverse: 5′ ACGGACTGAGGAGGAAACATGACAGTTGGAGGAAG 3′ [50]. In the sequence of the CASB forward primer, guanine (underlined) was substituted in place of cytosine, resulting in the creation of a restriction site for enzyme activity. The PCR mixture was initially prepared according to the manufacturer’s instructions for the KAPA Taq PCR Kit (Kapa Biosystems, Wilmington, MA, USA) and the recommended primer annealing temperature described by [50]. The ACRS-PCR assay used in the present study was adopted without modification from the protocol originally described by Olenski et al. [50], and all experimental procedures were performed according to the original methodological description. Subsequently, the composition of the PCR mixture was optimized to obtain clearer products on the electropherogram, as shown in Table 1.

A sufficient volume of the PCR mixture was prepared for the number of planned reactions, including one additional reaction as a negative control. The PCR thermal profile is presented in Table 2. To separate the restriction fragments, the PCR products were digested using the FastDigest Mph1103I enzyme (Thermo Fisher Scientific, Waltham, MA, USA) for 10 min at 37 °C, followed by enzyme inactivation at 65 °C for 15 min. The resulting PCR products were then visualized using gel electrophoresis.

### 2.6. Electrophoresis of the PCR Products

PCR products were visualized on an agarose gel (2.5%). A total of 5 μL of amplified DNA from each sample was mixed with 1 μL of 6× DNA Loading Dye buffer (Thermo Scientific, USA) and then carefully loaded into the wells of the gel using a pipette. Electrophoresis was carried out at a voltage of 100 V for 30 min using the Mupid-One apparatus (ADVANCE, Tokyo, Japan). Following electrophoresis, the agarose gels were post-stained with ethidium bromide and visualized under UV illumination using a UV transilluminator ETX-20-C (Vilber Lourmat, Collégien, France). This was performed following the standard procedure of the Laboratory for Animal Genetics, Department of Biology, Faculty of Veterinary Medicine, University of Belgrade.

### 2.7. Allele Frequency and Genotype Frequency

Genotype and allele frequencies (the frequency of genotypes and alleles) are the most important measures of genetic variation. Genotype frequency represents the percentage of individuals in a population that carry a specific genotype. This measure reflects the distribution of genetic variation within a population. Allele frequency represents the percentage of all copies of a given gene in a population that carry a specific allele. These measures accurately express the degree of genetic variation in a population. In the present study, separate analyses of genotype and allele frequencies were performed for HF cows and bulls, as well as for Busha cows.

### 2.8. Statistical Data Analysis

To conduct statistical analyses of β-casein gene polymorphism, allele frequencies, genotype frequencies, and their correlation with milk yield, milk protein content, and milk fat content in cows, the population’s genetic equilibrium was assessed using the Hardy–Weinberg principle and the χ^2^ test. Based on data homogeneity (CV < 30%) for milk production traits (milk yield and protein and fat content), groups were compared using a two-way ANOVA test with repeated measures for a single factor, followed by Tukey’s multiple comparison test between groups and Sidak’s multiple comparison test within groups. Statistical analyses of the study results were performed using the statistical software GraphPad Prism version 6 (GraphPad, San Diego, CA, USA).

## 3. Results

### 3.1. Genotyping Using ACRS-PCR

The DNA extracted from all cattle samples included in this study was amplified using the ACRS-PCR method (334 HF cows, 58 HF bulls, 64 Busha cows—a total of 456 samples). Before subjecting the samples to enzymatic digestion, they were analyzed by gel electrophoresis to confirm the success of the prior amplification.

**Figure 2:**
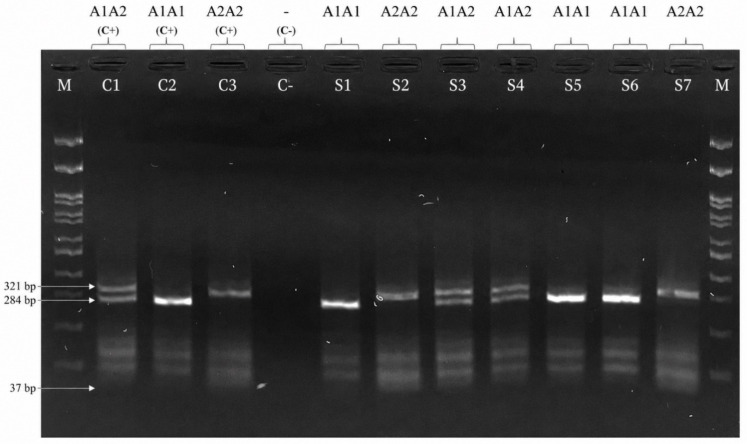
Analysis of PCR-amplified samples on agarose gel after enzymatic digestion using the ACRS-PCR method. M—DNA ladder (100 bp); C1–C3—positive controls; C− negative control; S1–S7—analyzed samples.

The amplified samples were digested using the Mph1103I enzyme, and these digested products were then analyzed by gel electrophoresis. Analysis of the electrophoretic bands on the agarose gel revealed three distinct genotypes: A1A1, A1A2, and A2A2. In the electropherogram shown in Figure 2, the following is observed: electrophoretic bands representing PCR products of 321 bp correspond to the A2A2 genotype; electrophoretic bands of 284 and 37 bp correspond to the A1A1 genotype; and electrophoretic bands representing PCR products of 321, 284, and 37 bp indicate the presence of the A1A2 genotype in the tested samples.

#### 3.1.1. Genotyping of the HF Breed Cows

**Figure 3:**
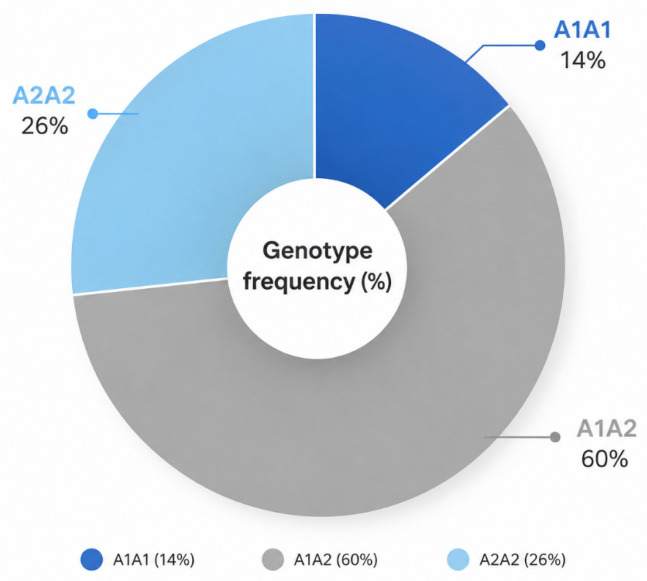
Genotype frequency of β-casein in HF cows.

In the population of 334 HF cows, 47 were of the A1A1 genotype, 201 of the A1A2 genotype, and 86 of the A2A2 genotype (Table 3 and Figure 3).

Accordingly, the observed genotype frequencies were A1A1—14.07%, A1A2—60.18%, and A2A2—25.75%. The allele frequency for A1 was 0.442, while for A2 it was 0.558. Based on these allele frequencies, the expected genotype frequencies under Hardy–Weinberg equilibrium were 19.54% for A1A1, 49.33% for A1A2, and 31.14% for A2A2, corresponding to expected counts of 65.25, 164.75, and 104.00, respectively. The chi-square test showed a significant deviation from Hardy–Weinberg equilibrium (χ^2^ = 16.19, df = 1, *p* < 0.001), indicating an excess of heterozygous A1A2 individuals.

#### 3.1.2. Genotyping of the HF Breed Bulls

**Figure 4:**
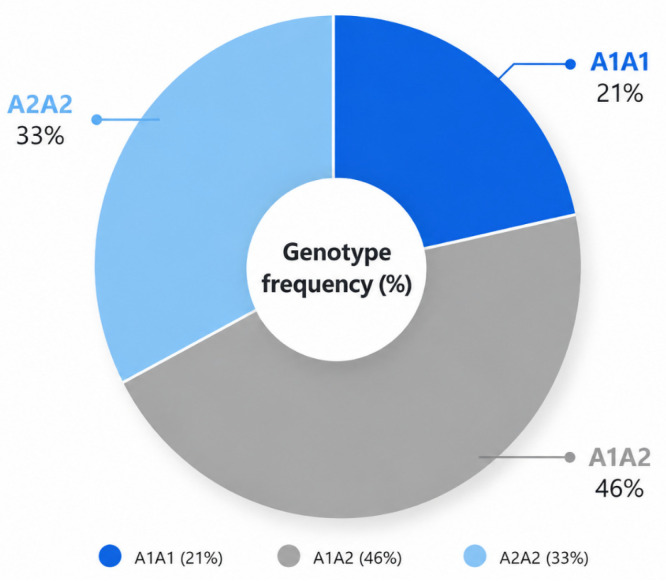
Genotype frequency of β-casein in HF bulls.

In the examined population of 58 HF bulls, 12 were of the A1A1 genotype, 27 of the A1A2 genotype, and 19 of the A2A2 genotype (Table 4 and Figure 4).

Accordingly, the observed genotype frequencies in HF bulls were A1A1—20.69%, A1A2—46.55%, and A2A2—32.76%. The allele frequency for A1 was 0.440, while for A2 it was 0.560. Based on these allele frequencies, the expected genotype frequencies under Hardy–Weinberg equilibrium were 19.36% for A1A1, 49.28% for A1A2, and 31.36% for A2A2, corresponding to expected counts of 11.23, 28.58, and 18.19, respectively. The chi-square test showed no significant deviation from Hardy–Weinberg equilibrium (χ^2^ = 0.177, df = 1, *p* = 0.674), indicating that the genotype frequencies were in accordance with Hardy–Weinberg equilibrium.

#### 3.1.3. Genotyping of the Busha Breed Cows

**Figure 5:**
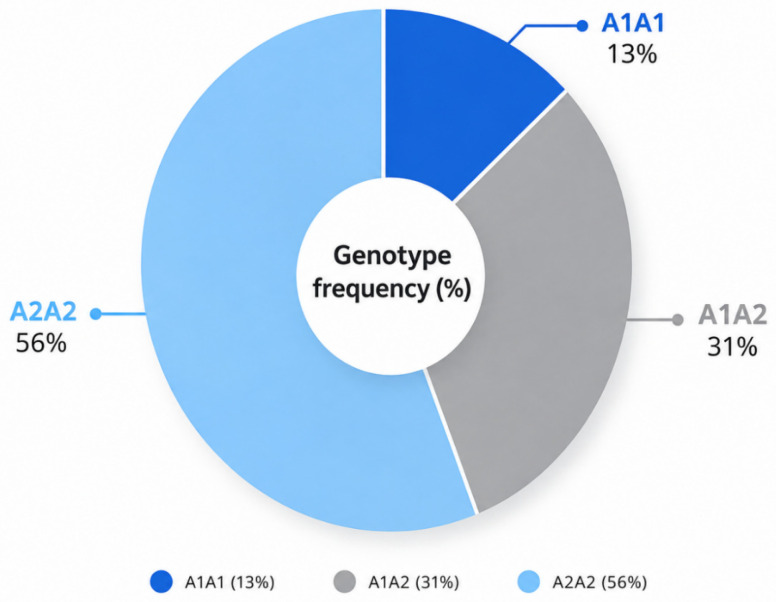
Genotype frequency of β-casein in Busha cows.

In the population of 64 Busha cows, eight were of the A1A1 genotype, 20 of the A1A2 genotype, and 36 of the A2A2 genotype (Table 5 and Figure 5).

Accordingly, the observed genotype frequencies in Busha cows were A1A1—12.50%, A1A2—31.25%, and A2A2—56.25%. The allele frequency for A1 was 0.281, while for A2 it was 0.719. Based on these allele frequencies, the expected genotype frequencies under Hardy–Weinberg equilibrium were 7.91% for A1A1, 40.43% for A1A2, and 51.66% for A2A2, corresponding to expected counts of 5.06, 25.88, and 33.06, respectively. The chi-square test showed no significant deviation from Hardy–Weinberg equilibrium (χ^2^ = 3.30, df = 1, *p* = 0.069), indicating that the genotype frequencies were in accordance with Hardy–Weinberg equilibrium.

### 3.2. Milk Yield, Milk Protein Content, and Milk Fat Content in HF Cows

#### 3.2.1. Milk Yield

**Figure 6:**
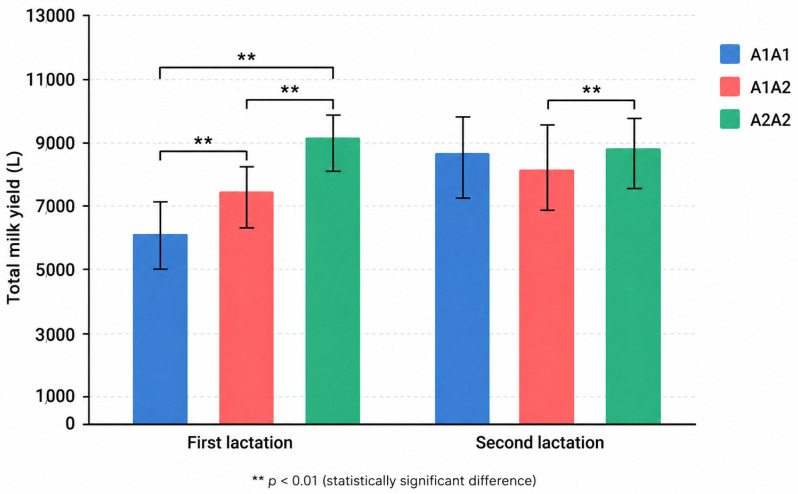
Milk yield in cows with different β-casein genotypes during the first and second lactation. ** *p* < 0.01.

**Figure 7:**
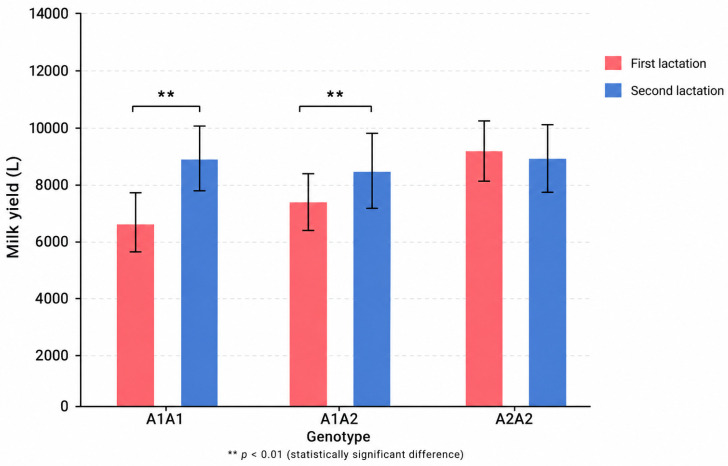
Comparison of milk yield between the first and second lactation in cows with the same β-casein genotypes. ** *p* < 0.01.

Analysis of milk production results in the first lactation revealed a significantly higher milk yield in A2A2 genotype cows compared to A1A1 and A1A2 genotype cows (*p* < 0.01), as well as in A1A2 cows compared to A1A1 genotype cows (*p* < 0.01; Figure 6). In the second lactation, a significantly higher milk yield was observed only in A2A2 genotype cows compared to A1A2 genotype cows (*p* < 0.01; Figure 6). Moreover, a significant increase in milk yield was observed in the second lactation compared to the first lactation for cows with the A1A1 and A1A2 genotypes (*p* < 0.01; Figure 7), but not for cows with the A2A2 genotype (*p* > 0.05; Figure 7). When comparing the total milk yield from both lactations (Table 6 and Supplementary Table S2), a statistically significantly higher milk production was found in A2A2 genotype cows compared to the other genotypes (*p* < 0.01), as well as in A1A1 genotype cows compared to A1A2 genotype cows (*p* < 0.05).

#### 3.2.2. Milk Protein Content

**Figure 8:**
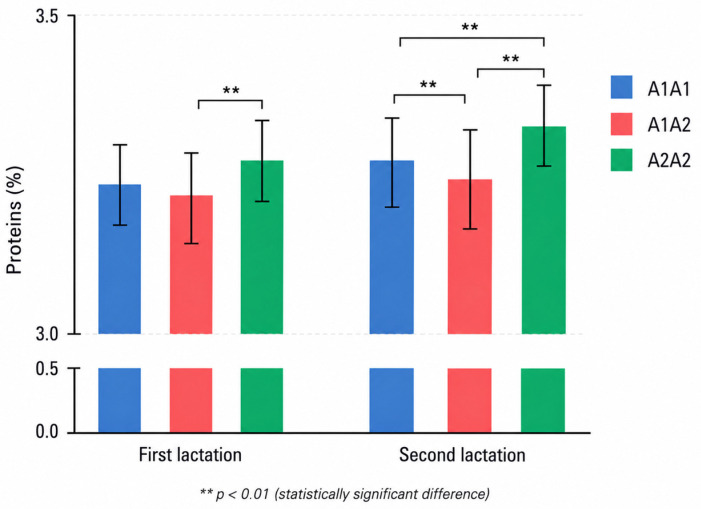
Milk protein content from cows with different β-casein genotypes during the first and the second lactation. ** *p* < 0.01.

**Figure 9:**
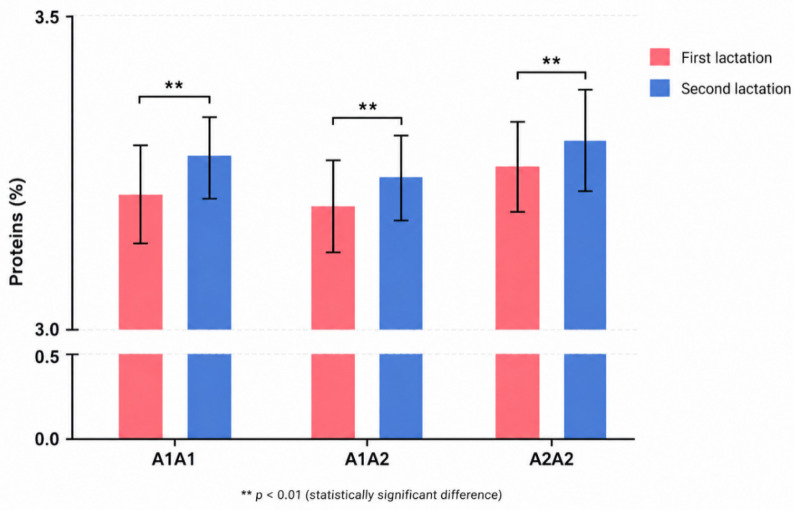
Comparison of milk protein content between the first and the second lactation in cows with the same β-casein genotypes. ** *p* < 0.01.

Analyzing milk protein content in cows with different β-casein genotypes, a statistically significantly higher protein percentage (*p* < 0.01; Figure 8) was observed in A2A2 genotype cows compared to A1A2 genotype cows. Statistical analysis of the results from the second lactation, as well as observing both lactations at the same time, showed the highest milk protein percentage in cows with the A2A2 genotype, which was statistically significantly higher compared to cows of the A1A2 genotype (*p* < 0.01) and the A1A1 genotype (*p* < 0.05; Figure 8). In addition, the milk protein content was significantly higher (*p* < 0.01) in A1A1 genotype cows compared to A1A2; in other words, A1A2 genotype individuals produced milk with the lowest protein content, which was statistically significant compared to the remaining genotypes (*p* < 0.01; Figure 8 and Supplementary Table S3). Statistical comparison of milk protein content between the first and second lactations revealed significantly higher values (*p* < 0.01; Figure 9) in the second lactation compared to the first, in all cows, regardless of their β-casein genotype.

#### 3.2.3. Milk Fat Content

**Figure 10:**
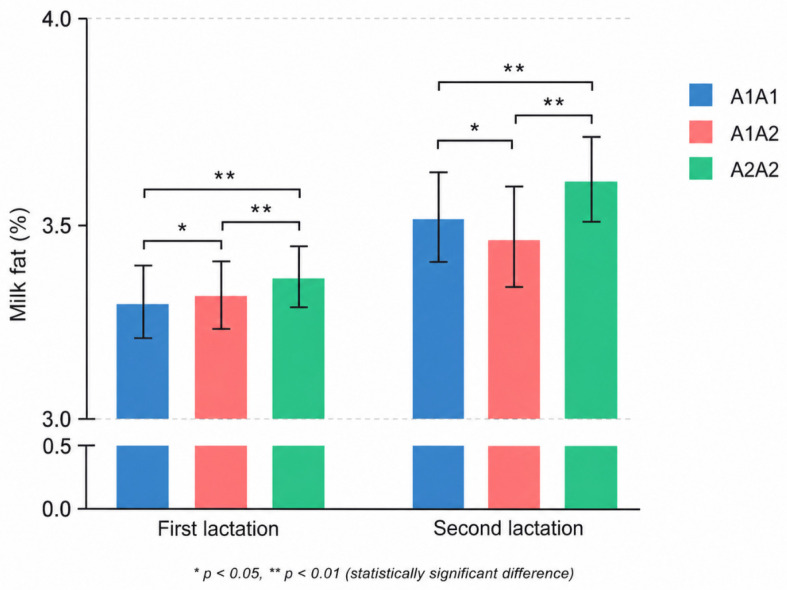
Comparison of milk fat from cows with different β-casein genotypes between the first and the second lactation. * *p* < 0.05, ** *p* < 0.01.

**Figure 11:**
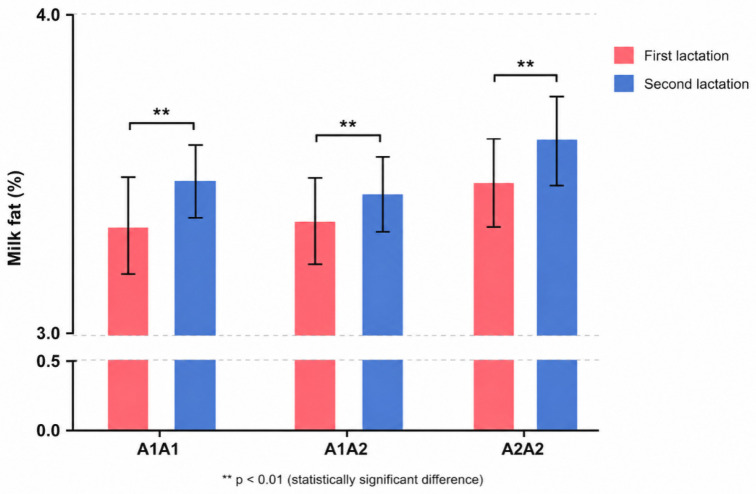
Comparison of milk fat content between the first and the second lactation in cows with the same β-casein genotypes. ** *p* < 0.01.

Milk fat content was highest in A2A2 genotype cows, and this difference was statistically significant (*p* < 0.01; Figure 10) compared to cows with other genotypes during separate lactations. Additionally, in the first lactation, higher milk fat values (*p* < 0.05; Figure 11) were detected in the milk of A1A2 genotype cows compared to milk of A1A1 genotype cows, while in the second lactation, the milk fat percentage was higher in A1A1 genotype cows compared to the milk of A1A2 genotype cows (*p* < 0.05; Figure 10). Comparing milk fat content between the first and second lactations, statistically significantly higher percentages (*p* < 0.01; Figure 11) were observed in the second lactation compared to the first lactation for all cows, regardless of β-casein genotype. Analysis of data on total secreted milk during both lactations showed that milk fat contents were statistically significantly higher in A2A2 genotype cows compared to the other cows in this study (*p* < 0.01; Table 6 and Supplementary Table S4).

## 4. Discussion

Large differences in β-casein allele frequencies have been reported among cattle populations worldwide. These are largely influenced by breeding strategies, genetic background, and the intensity of selection for milk production traits. In recent years, the A2 β-casein variant has attracted increasing scientific and commercial interest because of its potential importance for milk quality and human health. Allele distribution appears to depend on both breed origin and breeding history [5,51].

This study represents a substantial extension of our previous preliminary investigation conducted on 106 Holstein-Friesian cows [20]. By increasing the number of analyzed Holstein-Friesian cows and including Holstein-Friesian bulls and Busha cattle, we obtained a more comprehensive overview of β-casein polymorphism in Serbian cattle populations and improved the reliability of allele and genotype frequency estimates. The results obtained in HF cattle showed a higher frequency of the A2 allele in both cows (0.558) and bulls (0.560) compared to the A1 allele (0.442 and 0.440, respectively). Consequently, the heterozygous A1A2 genotype was the most frequent genotype in both examined HF populations. Similar results have been reported in several dairy cattle populations worldwide, such as in Polish HF cattle as reported by Kamiński et al. [19], Olenski et al. [27], and more recently by Kamiński and Cieślińska [18], where the A2 allele predominated over the A1 allele. Comparable results were also obtained in Italy [29,37,52], Greece [4], Slovakia [53], Denmark [33], and Taiwan [54]. The frequency of the A2 allele appears to be increasing in some HF due to the implementation of selective breeding programs favoring the A2A2 genotype and growing commercial interest in A2 milk production [44,46]. The similarity between our results and those reported in Central and Eastern European countries can probably be explained by the historical import of HF bulls and breeding material into Serbia from these regions, and the long-term use of related genetic lines in artificial insemination programs. In contrast, some populations from Northern Europe and North America still exhibit a relatively higher frequency of the A1 allele, which may reflect different breeding objectives and founder population effects [42,55].

Our findings in Busha cattle are especially interesting from the perspective of indigenous breed conservation. In our study, Busha cattle showed a pronounced predominance of the A2 allele (0.719) and the A2A2 genotype (56.25%), while the A1A1 genotype was detected at a relatively low frequency (12.50%). These findings are consistent with the hypotheses that the A2 allele represents the ancestral β-casein variant of β-casein and that indigenous breeds exposed to lower selection pressure retain a larger proportion of the original genetic structure [17,56]. These findings in Busha cattle are consistent with studies conducted in numerous indigenous and primitive cattle breeds worldwide, where the A2 allele generally predominates over the A1 allele. Dinc et al. [30] reported a predominance of the A2 allele in several Turkish indigenous cattle breeds, including Turkish Grey, Anatolian Black, Eastern Anatolian Red, and Southern Anatolian Red cattle. Similarly, Sodhi et al. [9] found a very high frequency of the A2 allele in the primitive Ladakhi cattle breed from India, while Gorkhali et al. [57] reported an almost complete predominance of the A2A2 genotype in indigenous Lulu cattle from Nepal. Comparable observations were also reported in Iranian native cattle breeds by Gholami et al. [35], particularly in Mazandarani and Taleshi cattle populations. Several European indigenous breeds have shown similar patterns: Ivanković et al. [58] analyzed β-casein polymorphism in Croatian Busha cattle and reported a predominance of the A2 allele and the A2A2 genotype, which is highly consistent with our findings. In the same study, the authors also examined two additional Croatian indigenous breeds, Istrian cattle and Slavonian–Syrmian Podolian cattle, both of which showed high frequencies of the A2 allele. Similar trends were observed in Greek local cattle breeds by Antonopoulos et al. [4], where the A2 allele was identified as the dominant β-casein variant in several indigenous populations adapted to extensive production systems. Studies conducted on native Italian cattle breeds, including Reggiana and Modenese cattle breeds, also demonstrated relatively high frequencies of the A2 allele compared to highly selected cosmopolitan dairy breeds [37,52]. Indigenous breeds from isolated or mountainous regions often show higher frequencies of the A2 allele. This is probably related to lower historical crossbreeding with modern high-yield dairy breeds and weaker artificial selection pressure. Indigenous breeds are generally maintained under extensive or semi-extensive production systems and are often characterized by greater genetic variability and preservation of ancestral alleles [59]. The predominance of the A2 allele in Serbian Busha cattle additionally supports the assumption that the A2 variant represents the original β-casein form that was widespread before intensive modern cattle breeding and crossbreeding programs contributed to the dissemination of the A1 allele in commercial dairy populations. Particularly important is the similarity between our results and those reported for Croatian Busha cattle by Ivanković et al. [58]. Considering the common breeding history of Busha populations throughout the Balkan Peninsula and the former Yugoslavia, as well as similar production systems and environmental conditions, such findings are expected. Nevertheless, the slightly higher frequency of the A2 allele observed in our Busha population may indicate a lower degree of historical crossbreeding with highly productive dairy breeds, such as Holstein-Friesian and Simmental cattle. Since Busha cattle in Serbia are still predominantly raised in extensive mountain production systems under relatively isolated conditions, they likely preserved a larger proportion of their original genetic structure.

Although these breeds are generally characterized by lower milk yield compared to modern commercial dairy breeds, their milk often possesses favorable compositional characteristics, including relatively high fat and protein contents [60,61], as well as potentially favorable β-casein profiles. The high frequency of the A2 allele observed in Busha cattle suggests that this indigenous breed may represent an important reservoir of genetic diversity for breeding strategies focused on increasing the prevalence of the A2 allele. However, because milk production traits were not evaluated in the Busha population, no conclusions can be drawn regarding its suitability as a production population for A2 milk programs.

In addition to genotype and allele frequency analysis, the present study investigated the association between β-casein genotypes and milk production traits in HF cows. Our results showed that cows carrying the A2A2 genotype achieved higher milk yield, as well as higher milk protein and milk fat contents compared to cows with other genotypes. Similar associations between the A2 allele and favorable milk production traits were previously reported by Nilsen et al. [62], Visker et al. [23], Olenski et al. [27], and more recently by Ardicli et al. [44]. The observed association may reflect the influence of linked genomic regions located within the casein gene cluster or elsewhere on BTA6. Recent genomic studies also confirmed that BTA6, where the CSN2 gene is located, contains numerous QTLs associated with milk yield and milk composition traits [49,63,64]. Thus, the observed differences between genotypes are probably influenced not only by β-casein polymorphism itself, but are also linked to genomic regions influencing dairy performance. In our study, cows with the A2A2 genotype consistently showed the highest production parameters during both lactations. Additionally, all examined cows achieved higher milk yield and improved milk composition during the second lactation regardless of genotype, which is consistent with the physiological maturation of dairy cows and increased mammary gland development during subsequent lactations. However, these findings should be interpreted with caution. Because the study design was observational and association-based, the results do not demonstrate a direct causal effect of CSN2 A1/A2 polymorphism on milk production performance. Interestingly, the relationship between β-casein genotype and milk composition traits did not appear to be strictly linear. Although cows carrying the A2A2 genotype generally exhibited the highest protein and fat concentrations, heterozygous A1A2 animals occasionally showed lower values than A1A1 cows. Similar observations have been reported in previous studies, suggesting that the effects of CSN2 polymorphism may not follow a simple allele–dose model. Milk production traits are complex quantitative characteristics influenced by various genetic and environmental factors, including nutrition, housing conditions, management, lactation stage, and physiological status of the animals [65,66]. Therefore, β-casein polymorphism should not be considered an isolated factor affecting dairy production. It should be emphasized that the present study identifies associations between β-casein genotypes and production traits and does not establish a direct causal relationship. The observed differences may reflect the functional effects of the CSN2 locus itself, linkage disequilibrium with neighboring genes within the casein cluster, or the influence of other loci located on BTA6. Consequently, additional studies involving larger populations and genome-wide approaches would be required to clarify the biological mechanisms underlying these associations. Still, our findings suggest that β-casein and other milk protein genotyping could be useful in breeding programs focused on improving both milk quality and dairy performance. A limitation of the present study is that β-casein genotypes determined by the ACRS-PCR assay were not independently confirmed by DNA sequencing or an alternative genotyping method. Although the applied protocol was adopted without modification from the method of Olenski et al. [50] and the obtained amplification and restriction patterns were consistent with the expected results, independent validation would further strengthen confidence in genotype assignment and will be considered in our future studies.

## 5. Conclusions

This study showed that the A2 allele was more frequent than the A1 allele in the examined HF cattle population, while the heterozygous A1A2 genotype was the most common. In the Busha population, a pronounced predominance of the A2 allele and the A2A2 genotype was observed, which suggests that this indigenous breed has retained much of its original genetic structure. Cows carrying the A2A2 genotype were associated with significantly higher milk yield, as well as higher protein and fat contents in both lactations compared to the other genotypes. In addition, the second lactation was characterized by higher milk production and increased protein and fat contents regardless of genotype. The observed results point to a favorable association of the A2A2 genotype with both milk yield and milk quality and could be useful for future breeding programs focused on A2 milk production. These findings may also contribute to future studies focused on the application of molecular genetics in sustainable cattle breeding.

## Figures and Tables

**Table 1:** Concentrations and volumes of reagents used for making the PCR mixture (ACRS-PCR).

Reagents	Stock Concentration	Final Concentration	25 µL Reaction
H_2_O	/	/	17.8 μL
10X KAPA Taq Buffer	10x	1x	2.5 µL
10 mM dNTP Mix	10 mM	0.2 mM	0.5 µL
Primer CASB F	20 µM	0.8 µM	1 µL
Primer CASB R	20 µM	0.8 µM	1 µL
5 U/µL KAPA Taq DNA Polymerase	5 U	1 U	0.2 µL
Template DNA	/	/	2 µL (100 ng)

**Table 2:** Themal cycling conditions for ACRS-PCR.

	Step	Temperature	Duration
	Initial denaturation	94 °C	3 min
35 cycles	Denaturation	94 °C	30 s
	Annealing	62.5 °C	30 s
	Elongation	72 °C	30 s
	Final elongation	72 °C	5 min

**Table 3:** Allele (A1/A2) and genotype frequencies of β-casein in Holstein-Friesian (HF) cows.

β-Casein Genotype	N	Genotype Frequency %	Allele Frequency	
			A1	A2
A1A1	47	14.07	0.442	0.558
A1A2	201	60.18		
A2A2	86	25.75		

**Table 4:** Allele (A1/A2) and genotype frequencies of β-casein in Holstein-Friesian (HF) bulls.

β-Casein Genotype	N	Genotype Frequency %	Allele Frequency	
			A1	A2
A1A1	12	20.69	0.440	0.560
A1A2	27	46.55		
A2A2	19	32.76		

**Table 5:** Allele (A1/A2) and genotype frequencies of β-casein in Busha cows.

β-Casein Genotype	N	Genotype Frequency %	Allele Frequency	
			A1	A2
A1A1	8	12.50	0.281	0.719
A1A2	20	31.25		
A2A2	36	56.25		

**Table 6:** Significance of influence of different β-casein genotypes on milk production characteristics (production in both lactations considered together). * *p* < 0.05, ** *p* < 0.01, ns = no significant difference.

Production Parameters	Tukey’s Multiple Comparison Test	Significance
Total milk production (L)	A1A1 vs. A1A2	*
	A1A1 vs. A2A2	**
	A1A2 vs. A2A2	**
Milk proteins (%)	A1A1 vs. A1A2	**
	A1A1 vs. A2A2	*
	A1A2 vs. A2A2	**
Milk fat (%)	A1A1 vs. A1A2	ns
	A1A1 vs. A2A2	**
	A1A2 vs. A2A2	**

## Data Availability

The original contributions presented in this study are included in the article/Supplementary Material. Further inquiries can be directed to the corresponding author.

## Supplementary Materials

The following supporting information can be downloaded at: https://www.mdpi.com/article/10.3390/ani16132052/s1, Figure S1: Schematic representation of the ACRS-PCR method for amplification of A1 and A2 β-casein alleles. Table S1: Allele frequencies of β-casein variants in indigenous and Holstein Friesian cattle breeds worldwide arranged chronologically by publication year. Table S2: Descriptive statistical analyses for milk yield. Table S3: Descriptive statistical analyses for milk protein content. Table S4: Descriptive statistical analyses for milk fat content.
