# Supplementary material for: β-Casein Polymorphism in Serbian Holstein-Friesian and Busha Cattle and Its Association with Milk Production Traits
Source: Animals (Basel). 2026 Jul 3;16(13):2052. doi: 10.3390/ani16132052 (PMC13359579; doi:10.3390/ani16132052)
Supplement: Supplementary file 1 [file animals-16-02052-s001.zip › Supplementary Figure S1.pdf]

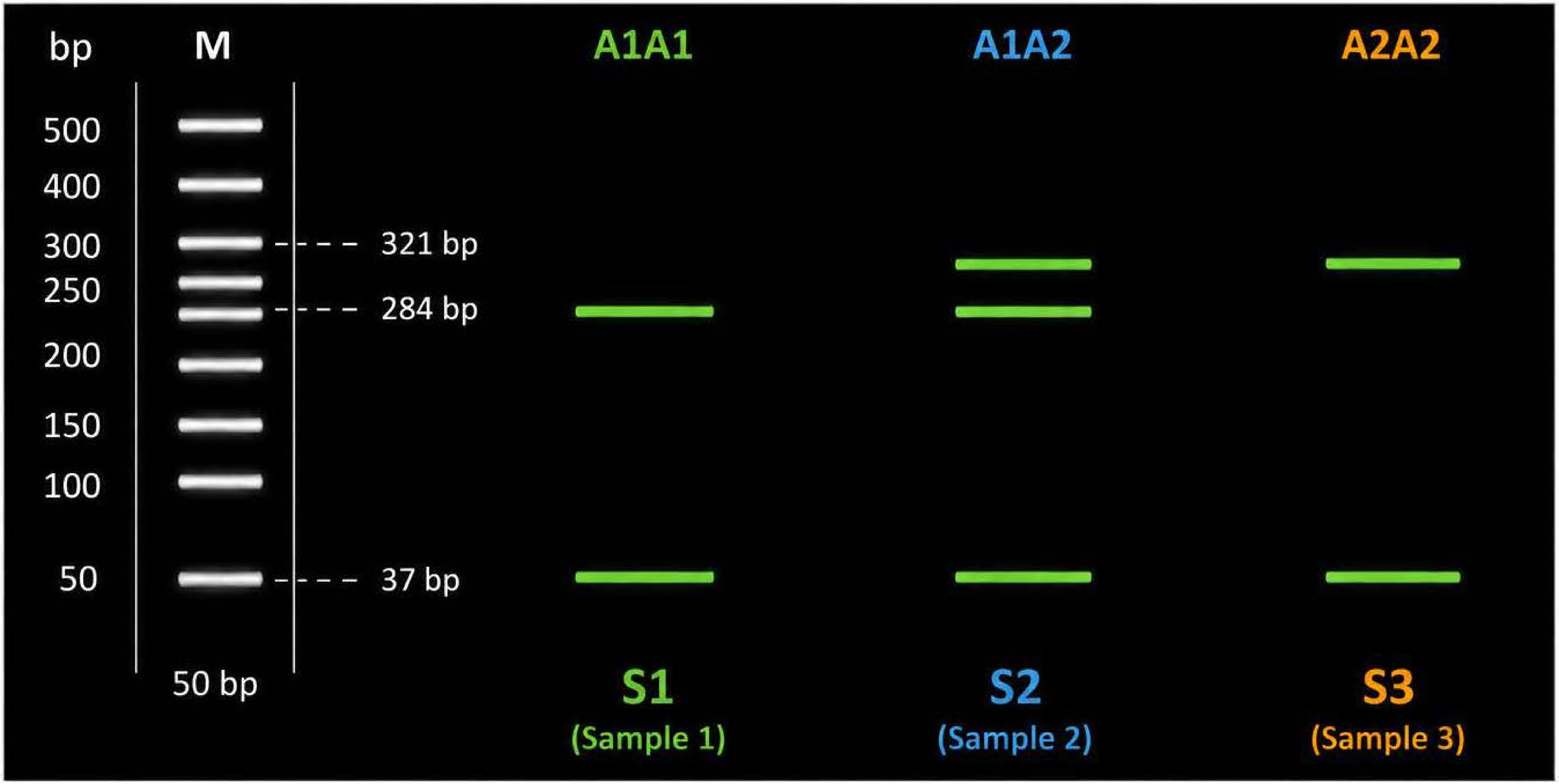

Supplementary Figure S1. Schematic representation of the ACRS-PCR method for amplification of A1 and A2 β-casein alleles.

M – DNA marker (50 bp)

S1 – A1A1 genotype of β-casein

S2 – A1A2 genotype of β-casein

S3 – A2A2 genotype of β-casein
