# Supplementary material for: β-Casein Polymorphism in Serbian Holstein-Friesian and Busha Cattle and Its Association with Milk Production Traits
Source: Animals (Basel). 2026 Jul 3;16(13):2052. doi: 10.3390/ani16132052 (PMC13359579; doi:10.3390/ani16132052)
Supplement: Supplementary file 1 [file animals-16-02052-s001.zip › Supplementary Table S1.pdf]

Supplementary Table S1. Allele frequencies of β-casein variants in indigenous and Holstein Friesian cattle breeds worldwide arranged chronologically by publication year

Year	Cattle breed	Country	A1 allele frequency	A2 allele frequency	Number of animals	Reference
2006	Holstein Friesian	Poland	0.402	0.598	143	Kaminski et al. 2006
2009	Holstein Friesian	Netherlands	0.285	0.692	1,912	Heck et al. 2009
2009	Gir	India	0.000	1.000	45	Mishra et al. 2009
2009	Sahiwal	India	0.000	1.000	47	Mishra et al. 2009
2009	Red Sindhi	India	0.000	1.000	33	Mishra et al. 2009
2010	Holstein Friesian	Netherlands	0.283	0.504	1.929	Visker et al. 2011
2010	Holstein Friesian	Czechia	0.450	0.550	120	Manga & Dvorak 2010
2011	Holstein Friesian	Thailand	0.363	0.602	231	Molee et al. 2011
2012	Holstein Friesian	Poland	0.320	0.680	177	Cieślińska et al. 2012
2012	Holstein Friesian	Poland	0.330	0.670	650	Olenski et al. 2012
2012	Vechur	India	0.20	0.80	72	Muhammed & Stephen 2012
2013	Holstein Friesian	Italy	0.395	0.530	100	Chessa et al. 2013
2013	Holstein Friesian	Turkey	0.485	0.456	49	Dinc et al. 2013
2013	Ongole	India	0.060	0.940	38	Ganguly et al. 2013
2014	Sahiwal	India	0.000	0.930	120	Mir et al. 2014
2014	Holstein Friesian	Denmark	0.266	0.614	415	Gustavsson et al. 2014
2016	Holstein Friesian	China	0.432	0.459	133	Dai et al. 2016
2016	Holstein Friesian	Iran	0.500	0.500	119	Gholami et al. 2016
2016	Malnad Gidda	India	0.014	0.986	119	Ramesha et al. 2016
2016	Kasargod Dwarf	India	0.042	0.958	48	Ramesha et al. 2016
2016	Deoni	India	0.000	1.000	40	Ramesha et al.

						2016
2016	Khillar	India	0.000	1.000	12	Ramesha et al. 2016
2017	Holstein Friesian	Italy	0.371	0.546	1.226	Massella et al. 2017
2017	Guzerá	Brazil	0.030	0.970	88	Rangel et al. 2017
2019	Tharparkar	India	0.040	0.960	81	Kumar et al. 2019
2019	Gir	India	0.184	0.816	226	Patel et al. 2019
2020	Holstein Friesian	Serbia	0.396	0.604	106	Ristanic et al. 2020
2021	Holstein Friesian	Greece	0.256	0.744	780	Antonopoulos et al. 2021
2021	Holstein Friesian	Germany	0.340	0.562	541	Meier et al. 2019
2021	Holstein Friesian	Russia	0.520	0.480	1081	Kovalyuk et al. 2021
2021	Greek Brachyceros (Vrachykerati ki)	Greece	0.304	0.587	46	Antonopoulos et al. 2021
2022	Polish Red	Poland	0.461	0.539	76	Barłowska et al. 2022
2022	Polish White-Backed	Poland	0.429	0.571	112	Barłowska et al. 2022
2022	Holstein Friesian	Poland	0.500	0.500	107	Barłowska et al. 2022
2023	Holstein Friesian	Turkey	0.320	0.680	1200	Ardicli et al. 2023
2023	Holstein Friesian	Slovakia	0.238	0.762	1478	Miluchova et al. 2023
2025	Holstein Friesian	Ecuador	0.372	0.628	701	Cartuche-Macas et al. 2025
2025	Ecuadorian Gyr	Ecuador	0.145	0.855	258	Cartuche-Macas et al. 2025
2025	Polish White-Backed	Poland	0.388	0.612	777	Sawicka-Zugaj et al. 2025
