# Supplementary material for: β-Casein Polymorphism in Serbian Holstein-Friesian and Busha Cattle and Its Association with Milk Production Traits
Source: Animals (Basel). 2026 Jul 3;16(13):2052. doi: 10.3390/ani16132052 (PMC13359579; doi:10.3390/ani16132052)
Supplement: Supplementary file 1 [file animals-16-02052-s001.zip › Supplementary Table S2.pdf]

**Supplementary Table S2**. Descriptive statistical analyses for milk yield.

Descriptive statistics for milk yield according to β-casein genotype and lactation.

| Lactation | A1A1 | A1A2 | A2A2 |
|---|---|---|---|
| | mean ± SD | mean ± SD | mean ± SD |
| First lactation | 6443.00±1135.00 | 7670.00±1080.00 | 9336.00±735.20 |
| Second lactation | 8974.00±1645.00 | 8640.00±1560.00 | 9217.00±1366.00 |

Results of two-way ANOVA with repeated measures for milk yield.

| Effect | F (DFn, DFd) | P-value | partial $\eta^2$ |
|---|---|---|---|
| Genotype | F(2, 331) = 45.72 | < 0.0001 | 0.217 |
| Lactation | F(1, 331) = 131.30 | < 0.0001 | 0.284 |
| Genotype × lactation | F(2, 331) = 46.77 | < 0.0001 | 0.220 |
