# Supplementary material for: β-Casein Polymorphism in Serbian Holstein-Friesian and Busha Cattle and Its Association with Milk Production Traits
Source: Animals (Basel). 2026 Jul 3;16(13):2052. doi: 10.3390/ani16132052 (PMC13359579; doi:10.3390/ani16132052)
Supplement: Supplementary file 1 [file animals-16-02052-s001.zip › Supplementary Table S3.pdf]

Supplementary Table S3. Descriptive statistical analyses for milk protein content.

Milk protein content (%) according to β-casein genotype and lactation.

Lactation	A1A1	A1A2	A2A2
	mean ± SD	mean ± SD	mean ± SD
First lactation	3.11±0.05	3.10±0.07	3.14±0.10
Second lactation	3.20±0.05	3.16±0.08	3.24±0.07

Results of two-way ANOVA with repeated measures for milk protein content.

Effect	F (DFn, DFd)	P-value	partial η^2
Genotype	F(2, 331) = 30.04	< 0.0001	0.154
Lactation	F(1, 331) = 186.30	< 0.0001	0.360
Genotype × lactation	F(2, 331) = 4.95	0.0076	0.029
