# Supplementary material for: β-Casein Polymorphism in Serbian Holstein-Friesian and Busha Cattle and Its Association with Milk Production Traits
Source: Animals (Basel). 2026 Jul 3;16(13):2052. doi: 10.3390/ani16132052 (PMC13359579; doi:10.3390/ani16132052)
Supplement: Supplementary file 1 [file animals-16-02052-s001.zip › Supplementary Table S4.pdf]

Supplementary Table S4. Descriptive statistical analyses for milk fat content.

Descriptive statistics for milk fat according to β-casein genotype and lactation.

Lactation	A1A1	A1A2	A2A2
	mean ± SD	mean ± SD	mean ± SD
First lactation	3.27±0.10	3.31±0.08	3.37±0.08
Second lactation	3,45±0.05	3.41±0.10	3.52±0.10

Results of two-way ANOVA with repeated measures for milk fat.

Effect	F (DFn, DFd)	P-value	partial η^2
Genotype	F(2, 331) = 46.52	< 0.0001	0.219
Lactation	F(1, 331) = 450.70	< 0.0001	0.577
Genotype × lactation	F(2, 331) = 13.14	< 0.0001	0.074
